# Construct and predictive validity of the Strength of Motivation for Medical School-Revised (SMMS-R) questionnaire: a French validation study

**Published:** 2019-07-24

**Authors:** Milena Abbiati, François Severac, Anne Baroffio, Thierry Pelaccia

**Affiliations:** 1 Unit of Development and Research in Medical Education (EDREM), University of Geneva, Faculty of Medicine, Switzerland; 2 Strasbourg University Hospital, France; 3 Center for Training and Research in Health Sciences Education (CFRPS), University of Strasbourg, Faculty of Medicine, France

## Abstract

Motivation is a major indicator of students’ learning behaviors. Therefore, researchers require consistent and valid instruments to assess students’ motivation. Consequently, motivation has been an important topic in medical education research for the last decade. The present study evaluated the construct and predictive validities of the French version of the Strength of Motivation for Medical School-Revised questionnaire (SMMS-R-FR). Our sample comprised 372 students at three French-speaking medical schools, who filled in the SMMS-R-FR and the Revised two-factor Study Process Questionnaire (R2-SPQ). Results confirmed the three-factor structure of the original SMMS-R questionnaire. Reliabilities were good for the Total Strength of Motivation scale, moderate for the Willingness to Sacrifice and Readiness to Start subscales, and poor (but still acceptable) for the Persistence subscale. Both Total Strength of Motivation and Readiness to Start positively predicted a deep learning approach and negatively predicted a surface learning approach, while Willingness to Sacrifice positively predicted a deep learning approach and Persistence negatively predicted a surface learning approach. Our results both support the SMMS-R- FR’s suitability as a tool for measuring motivation in medical students, and suggest that it could be used to guide the development of educational interventions to strengthen motivation.

## Introduction

Motivation, defined as “any internal process that energizes, directs, and sustains behavior,”^1^ is the driving force that underlies “why people think and behave as they do.”^2^ Studies of motivation’s impact on training date back to the 1930s, but higher education researchers, especially those in the field of health sciences, did not start taking an interest in the issue until much later, as they assumed that students who choose their field of study must necessarily be motivated.^3,4^ However, research on medical education has highlighted the importance of motivation in training, with higher levels of student motivation predicting engagement in learning activities; perseverance, even in the case of failure; mobilization of effective learning approaches; and academic performance.^5-11^ Given the degree to which motivation affects medical training, White and Gruppen called upon researchers to make motivation a key topic in medical education studies.^12^ To do this, researchers must have consistent, reliable, and valid instruments for measuring student motivation.^13^

Many of the instruments commonly used to study motivation in medical education were inspired by Self-Determination Theory^4,14^ and most are designed to assess types of motivation, that is whether an individual is motivated to act by external factors ("extrinsic motivation") or by internal factors ("intrinsic motivation"). These instruments include the Self-Regulation Questionnaire (SRQ) and the Academic Motivational Scale (AMS).^15-19^

The Strength of Motivation for Medical School (SMMS) questionnaire was developed in the Netherlands in the early 2000s to assess the strength of motivation, defined as “the applicant's or student's readiness to start and continue medical training regardless of sacrifices, setbacks, misfortune or disappointing perspectives.”^20^ As the authors highlighted, strength of motivation does not necessarily correlate with quality of motivation. Responses to each of the SMMS’s sixteen items, noted on 5-point Likert scales, are combined to give a single Total Strength of Motivation score. However, the authors warned against using SMMS assessments for high-stake selection purposes because of the risk of a social-desirability effect on students’ responses. Instead, they recommended using the SMMS throughout the medical school program in order to assess the ability of selection processes to identify the best equipped students (e.g., those who are the most highly motivated).

A subsequent validation study led to a revised version of the SMMS (SMMS-R) containing fifteen items, which were divided into three subscales on the basis of a principal components analysis. Hence, the SMMS-R provides three subscale scores as well as a Total Strength of Motivation score.^21^ The SMMS-R has acceptable reliability and correlates moderately positively with the AMS and negatively with Maslach Burnout Inventory-Student Survey Exhaustion subscale.^6,22^ These correlations are in line with constructs measured by AMS and Maslach’s Exahustion subscale: motivation in students and feeling exhausted, respectively. In addition, SMMS- R scores are linked to the way students gain entry to medical school, with students admitted following a selection procedure obtaining higher Total Strength of Motivation scores than those admitted via a lottery or on the basis of school examination grades.^23,24^ Finally, strength of motivation is a dynamic parameter that primarily due to the age and maturity and, to a small extent, with gender, older female students showing the highest SMMS-R scores.^22^

Nevertheless, more research is needed to confirm the SMMS-R’s construct and predictive validities with respect to assessing motivation. To the best of our knowledge, An et al. is the only study to have analyzed the validity and accuracy of the SMMS-R outside the Netherlands.^25^ Their findings, based on a sample of 986 Chinese medical students, support the SMMS-R’s three-factor structure. The present study further extends these validity analyses by focusing on French-speaking countries in order to allow SMMS-R use in their Medical schools and therefore provide a consistent instrument for motivation assessment. Moreover, previous studies have explored how the type of motivation influences learning approaches, but have not analyzed specifically how the strength of motivation affects learning approaches. Conclusive findings could or could not support educational intervention to strengthen motivation which in turn would optimize learning approaches. To this end, we 1) evaluated the reliability of the three-factor structure of the French version of the SMMS-R (Force de la Motivation chez les Etudiants en Médecine, SMMS-R-FR) and 2) determined the SMMS-R-FR’s predictive validity for deep and surface learning approaches.

## Methods

### Survey Setting

The present study was part of a larger, multi-site research project that follows cohorts of medical students throughout their training program, assessing, among other things, the impact of motivation on learning strategies, academic performance, and career choices.

### Sample and survey administration

Our multi-site, cross-sectional study focused on three French-speaking medical schools in Switzerland (Lausanne, Geneva) and France (Strasbourg). All three schools offer a full, six-year medical training program that includes a student-selection process, based on an examination taken at the end of the first year (pass rates are around 40% in Switzerland and 30% in France).

Before beginning our study, we obtained approval for the survey from Switzerland’s Institutional Review Board and France’s Research Ethics Board. We collected data at all three medical schools in May 2018. Students (Year 3 in Lausanne, Year 5 in Geneva and Strasbourg) completed the survey on a voluntary basis during a regular class period. Responses were anonymous. We produced both paper and online versions of the survey, so students who had their own computers in the classroom could provide their responses online. In order to include students who were not in class when we administered the paper questionnaire, we sent reminders to complete the online version one and two weeks after it was first administered.

Average return rates for the Lausanne (LA), Geneva (GE), and Strasbourg (STR) medical schools were 74% (N= 104/140), 96% (N= 84/88), and 98% (N=189/192), respectively, giving an overall mean return rate of 90% (N= 377/420). We excluded five students (one male in LA, one female in GE, two males and one female in STR) from our analyses because they answered less than 85% of the items on the questionnaire, so our final sample comprised 372 students.

Considering all three schools, 63% of the respondents were female (n=235). As we expected, given that LA students were in Year 3 and GE and STR students were in Year 5, respondents from LA (mostly born in 1995) were significantly younger than respondents at GE and STR (mostly born in 1995 and 1994, respectively, p<.001).

### Measures

***Strength of Motivation for Medical School - Revised - French version (SMMS-R-FR)*.** The SMMS-R is a structured professional instrument for evaluating strength of motivation for medical studies.^21^ The scale’s 15 items, five of which are reversed (R), are scored on 5-point Likert scales (1 = *strongly disagree* to 5 = *strongly agree*). These 15 items are divided into three subscales, each containing five items: Willingness to Sacrifice (e.g., “Even if I could hardly maintain my social life, I would still continue medical training”), Readiness to Start/Continue (e.g., “I wouldn’t consider any other profession than becoming a doctor”), and Persistence (e.g., “I would quit studying medicine if I were 95% certain that I could never become a specialist in the field of my choice”). Summing the scores for the three subscales gives a Total Strength of Motivation score. Subscale scores can range from 5 to 25, so total scores can range from 15 to 75. We used the French version of the SMMS-R (Force de la Motivation chez les Etudiants en Médecine, SMMS-R-FR), which two independent reviewers at our institutions had translated from English to French and then translated back into English through an iterative process which follows a backward translation.

***Revised two-factor Study Process Questionnaire (R2- SPQ)*.** The R2-SPQ^26^ is widely used to assess two major, non-exclusive learning approaches: the Deep Approach and the Surface Approach. It comprises twenty items scored on 5-point Likert scales (1 = *This item is never or only rarely true of me* to 5 = *This item is always or almost always true of me*). Total scores for both approaches can range from 10 (rare) to 50 (always). Learners with high Deep Approach scores try to understand what they are studying and to relate ideas to previous knowledge and experience. Learners with high Surface Approach scores memorize facts and figures in order to pass exams, and expend the least possible effort to accomplish what is required. We decided to use Gustin’s^27^ version of the R2-SPQ, without any modification, because of the strong theoretical basis of the instrument and the satisfactory 20-item Cronbach’s alpha (α=.73 for both Deep Approach and Surface Approach).

## Data Analysis

### Descriptive

We calculated descriptive statistics for demographics, SMMS-R-FR, and R2-SPQ. The Chi-square was used to compare the proportions of female and male respondents at each school. We also conducted ANOVAs with *post hoc* Bonferroni corrections to compare the ages of respondents at each school and to determine whether there were any differences between the schools in terms of SMMS-R-FR total scale and subscale scores, and R2-SPQ Deep Approach and R2-SPQ Surface Approach scores.

We used the classic Cronbach’s alpha coefficients to assess reliability. Critical values for single measures were: α > .90 = excellent, α > .80 < .90 = good, α > .70 < .80 = acceptable, α > .60 < .70 = questionable, α > .50 < .60 = poor, and α <.50 = unacceptable.^28^ Type I error rates were set at .01.

### Construct validity

Because we hypothesized that factors are dependent on each other, we used a principal component analysis (PCA) with Promax Rotation to aggregate the 15 SMMS-R-FR items. We checked the correlations between items (Bartlett’s test) as well as multicollinearity. We combined criteria (i.e., scree plot, eigenvalue < 1.5, and interpretability) to check the three-factor structure of the original, English version of the SMMS-R^29^ and calculated Pearson’s correlations between retained factors. Critical values for Pearson’s r correlations were: r > .50 is high; .50 > r > .30 is moderate, and .30 > r > .25 is low.^30^ The critical value for significant factor loading was > .40.^31^

### Predictive validity

We used linear regression analyses (mean difference and 95% confidence intervals) to determine whether SMMS-R-FR total scale and subscales predicted Deep Learning and Surface Learning approaches, as reported in previous studies of motivation and learning approaches.^7,8^ We checked the usual linear regression assumptions (normality, homoscedasticity, autocorrelation of residuals and multicollinearity).

We used SPSS version 25 for all our analyses (IBM Corp., Armonk, NY, USA).

## Results

### Descriptive

Table 1 shows the means of the SMMS-R-FR total and subscale scores, and 2R-SPQ scores for each school.

**Table 1:** SMMS-R-FR and R2-SPQ descriptive statistics by school

Measures	Mean [Range]	All N = 372	Lausanne n = 103	Site Geneva n = 83	Strasbourg n=186	ANOVA p value
**SMMS-R-FR**	WS	15.2 [5-25]	15.7 [7-24]	14.7 [5-22]	15.1 [5-25]	.110
	RS	16.4 [5-25]	16.2 [7-25]	16.4 [7-25]	16.5 [5-25]	.717
	P	16.6 [7-51]	17.0 [8-25]	15.9 [7-22]	16.8 [7-24]	.205
	TSM	48.2 [7-59]	48.9 [8-50]	46.9 [7-59]	48.4 [7-51]	.287
**R2-SPQ**	DA	28.9 [13-47]	28.2 [13-44]	28.7 [15-42]	29.5 [15-47]	.270
	SA	22.9 [10-43]	24.5 [11-40]	22.1 [11-40]	22.3 [10-43]	**.014**

Note. SMMS-R-FR = Strength of Motivation for Medical School-Revised-French Version; WS = Willingness to Sacrifice; RS= Readiness to Start; P = Persistence; TSM = Total Strength of Motivation; R2-SPQ = Revised two-factor Study Process; DA = Deep Approach; SA = Surface Approach

There were no differences between the schools in the means for the SMMS-R-FR total scale and subscale scores. However, the mean Surface Approach score on the 2R-SPQ was statistically significantly higher for students from LA than for students from GE and STR (p < .01).

As Table 2 shows, the reliability of the SMMS-R-FR was acceptable for Total Strength of Motivation and questionable to acceptable for the Willingness to Sacrifice and Readiness to Start subscales for all the schools. The reliability of the Persistence subscale was poor. Cronbach’s alphas for this subscale were lowest for GE, but still within acceptable limits. Reliability of the 2R-SPQ was acceptable for both the Deep Approach and the Surface Approach for all three schools.

**Table 2:** SMMS-R-FR and R2-SPQ reliability by school

Measures/Cronbach’s alpha		All (N = 372)	Lausanne (*n* = 103)	Geneva (*n* = 83)	Strasbourg (*n* = 186)
**SMMS-R-FR**	WS	.67	.64	.64	.69
	RS	.68	.74	.68	.70
	P	.56	.58	.55	.58
	TSM	.78	.79	.76	.80
**R2-SPQ**	DA	.73	.71	.74	.74
	SA	.73	.70	.70	.74

Note. SMMS-R-FR = Strength of Motivation for Medical School-Revised-French Version; WS = Willingness to Sacrifice; RS= Readiness to Start; P = Persistence; TSM = Total Strength of Motivation; R2-SPQ = Revised two-factor Study Process; DA = Deep Approach; SA = Surface Approach

### Construct validity

**Figure 1:**
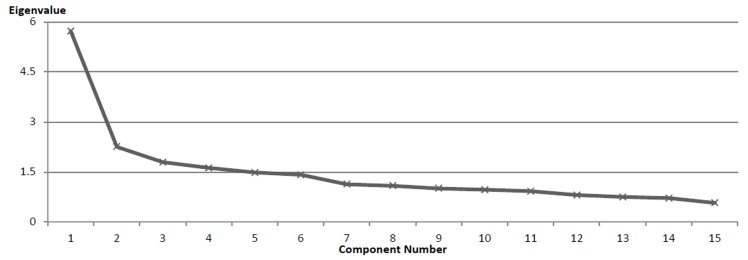
Screen plot of PCA eigenvalues for the SMMS-R-FR

Figure 1 shows the PCA with Promax Rotation scree plot for the SMMS-R-FRS, with no constraints on the number of factors. The scree method gave a critical angle at Factor 2 and, to a lesser extent, after Factor 3 and after Factor 4.

The PCA yielded three factors (KMO .88, p<.001, 44% of variance explained, see Table 3). Factor 1, combining items 5, 7, 9, 10, & 12, explained 26% of the variance. Factor 2, combining items 1, 3, 6, 11, & 15, was responsible for 20% of the variance. Factor 3, combining items 2, 4, 8, 13, & 14, accounted for 8% of the variance.

**Table 3:** The SMMS-R-FR’s three component factors as revealed by PCA with promax rotation (KMO = .88, p < .001, 43.9% of variance explained)

	Factor loadings		
Items	F1	F2	F3
**#5**	**.743**	.322	.234
**#7**	**.699**	.284	.176
**#10**	**.595**	.292	.331
**#12**	**.591**	.238	.048
**#9**	**.439**	.130	.334
**#15**	.177	**.789**	.371
**#6**	***.467***	**.680**	.141
**#1**	.193	**.652**	-.060
**#3**	***.486***	**.549**	.193
**#11**	***.453***	**.521**	.348
**#14**	.330	.255	**.695**
**#13**	.167	.102	**.641**
**#4**	***.519***	.202	**.558**
**#8**	.305	.083	**.520**
**#2**	-.081	.080	**.479**
	**% Variance explained**		
	**F1**	**F2**	**F3**
	25.774	10.153	8.053
	**Factor correlations**		
	**F1**	**F2**	**F3**
**F1**	1.000		
**F2**	.403	1.000	
**F3**	.336	.250	1.000

Items 3, 4, 6, and 11 cross-loaded significantly (> .40) on Factor 1, but cross-loadings on this second factor were always lower than the cross-loadings on their primary factor.

Factor 1 was positively and moderately significantly correlated with both Factors 2 and 3. We also found a low, but still significant, positive correlation between Factor 2 and Factor 3.

### Predictive validity

Linear regression analyses of students’ learning approaches showed that Willingness to Sacrifice and, to a lesser extent, Readiness to Start have positive predictive validity for Deep Approach (see Table 4). Conversely, Persistence and, to a lesser extent, Readiness to Start have negative predictive validity for Surface Approach.

**Table 4:** Linear regression analysis of the SMMS-R-FR total scale’s and subscales’ predictive validities for the deep and surface approaches

SMMS-R-FR	Deep Approach Mean difference [95% CI]	p	Surface Approach Mean difference [95% CI]	p
	R ^2^=.194 (r=441)		R ^2^=.075 (r=288)	
**Willingness to sacrifice**	.440 [ .3 ; .6]	**.000**	-.037 [- .2 ; -.1]	.700
**Readiness to start**	.269 [ .2 ; .4]	**.000**	-.223 [- .4 ; -.1]	**.005**
**Persistence**	.119 [ .1 ; .1]	.155	-.300 [- .5 ; -.1]	**.001**
	R ^2^=.182 (r=427)		R ^2^=.074 (r=274)	
**Total**	.281 [ .2 ; .3]	**.000**	-.187 [- .2 ; -.1]	**.000**

In addition, Total Strength of Motivation has positive predictive validity for Deep Approach and negative predictive value, although with lower Beta values, for Surface Approach.

## Discussion

The present study applied the French version of the SMMS-R (SMMS-R-FR) to three classes of medical students in French-speaking countries, in order to assess the scale’s construct and predictive validities for deep and surface learning approaches. Over all, results confirm positive similarity between our validation and the original version. Results confirmed the reliability and validity of the SMMS-R’s original three-factor structure. In addition, predictive validity was good for the Total Strength of Motivation scale and for all three of the SMMS-R-FR’s subscales, although correlation strengths varied across scales and learning approaches.

Our work confirms the SMMS-R-FR’s internal reliability, which, similarly to the original validation, was moderate to good for all the scales except the Persistence subscale. As for the original SMMS-R, the reliability of the Persistence subscale was poor, but still acceptable.^21^ Hence, the SMMS-R-FR’s Total Strength of Motivation scale and three subscales can be considered suitable for use with French-speaking medical students. However, results for the Persistence subscale must be interpreted with caution.

In terms of the scale’s construct validity, our results confirm the original SMMS-R’s three-factor structure. Combining psychometric criteria with existing theory (the three-factor structure reported by Kusurkar et al., 2011)^3^ and the interpretability of the items’ content provided further support for this structure.^29^ However, in contrast with the SMSS-R, cross-loadings between factors were significant for some of the items (Pearson’s r > .40), most notably for some Factor 2 items, which cross-loaded onto Factor 1. This is consistent with the high (r = .49) correlation between these two factors.

Results showed significant predictive validities for all three of the SMMS-R-FR’s subscales, as well as for the Total Strength of Motivation scale. Significance levels were highest for Willingness to Sacrifice and Persistence, but for just one of the two learning approaches (positive mean difference between Willingness to Sacrifice and Deep Approach, negative mean difference between Persistence and Surface Approach). Readiness to Start and, to a lesser extent, Total Strength of Motivation significantly predicted both learning approaches (positive mean difference with Deep Approach, negative correlations with Surface Approach). Predictive validity was weaker, but still significant, for the Total Strength of Motivation scale. Hence, as for the original SMMS, it is best not to use Total Strength of Motivation scores on their own.

### Conclusion

Overall, SMMS-R-FR scores positively predicted a deep approach and negatively predicted, but to a lesser extent, a Surface Approach. This suggests that strengthening motivation in students could be a way of fostering a deep learning approach and thereby improving academic performance. Promoting a deep learning approach is a huge challenge in medical education.^32,33^ Evidence for the impact of contextual factors on the choice of learning approach is limited and sometimes contradictory.^34-37^ Our findings about the SMMS-R-FR’s predictive validity complement studies into the impact of motivation on learning approaches^3^ and pave the way for developing new educational interventions aimed at strengthening motivation.

Nevertheless, because our study involved a relatively small sample of medical students, its findings may not be generalizable to the SMMS-R, and further validation is needed to confirm the superiority of the SMMS-R’s three-factor structure over the original one-factor solution. In addition, all the students in our sample were French speakers and had gone through similar medical school selection procedures (based on multiple-choice questionnaires at the end of year 1). Further studies are needed to validate the SMMS-R and compare results for other populations of students. A larger and more diverse sample could be used to identify cut-off points at which SMMS-R scores predict various individual and contextual variables (e.g., educational context, type of motivation, academic achievement). Finally, our study’s cross-sectional design prevented us investigating the stability of SMMS-R-FR scores and how motivation varies as students progress through medical school. We are currently conducting a follow- up study in the three medical schools.

We recommend using the SMMS-R-FR in French- speaking medical schools because we believe it has an important role to play in assessing student motivation. However, like the SMMS-R, the SMMS-R- FR is not intended to be used as a selection tool, but it can be used to assess how well a selection process works or to better understand the characteristics of a cohort of medical students. The SMMS-R-FR could also be used to guide the development of educational interventions to improve motivation. If our results are confirmed and extended by further analysis, this could be a way to increase deep learning strategies among medical students.

## Appendix

The SMMS-R Questionnaire-French version

Chacun a des raisons différentes pour étudier la médecine. Indiquez svp combien les énoncés suivants s'appliquent à votre situation personnelle en utilisant une échelle en 5 points (1= pas du tout d'accord; 5 = tout à fait d'accord)

**Table:**

n	SMMS-R item	Sous-echelle	Score inverse
1.	Je regretterais toujours ma décision si je ne m'étais pas donné la chance d'étudier la medicine	2	
2.	J’arrêterais d’étudier la médecine si j’étais à 95% sûr de ne jamais pouvoir pratiquer dans la spécialité de mon choix	3	INV
3.	Je choisirais de toute façon la médecine, même si cela impliquait d’étudier dans un pays étranger, dans une langue que je ne maîtrise pas encore	2	
4.	Si je découvrais qu’il me fallait encore dix ans pour obtenir le diplôme de médecin, j’arrêterais mes etudes	3	INV
5.	Même si je pouvais difficilement conserver une vie sociale, je continuerais toujours ma formation médicale	1	
6.	Je ne prendrais en considération aucune profession autre que devenir un médecin	2	
7.	Je choisirais toujours la médecine, même si cela impliquait que je ne serais jamais plus en mesure de partir en vacances avec mes amis	1	
8.	J'arrêterais d’étudier la médecine si je commençais à obtenir des mauvaises notes et à échouer souvent aux épreuves	3	INV
9.	Si étudier me prenait plus que 60 heures en moyenne par semaine, je réfléchirais sérieusement à arrêter	1	INV
10.	J’ai l’intention de devenir un médecin, même si cela impliquait de suivre des cours en formation continue deux soirs par semaine tout au long de ma carrière professionnelle	1	
11.	Cela ne me dérangerait vraiment pas beaucoup si je ne pouvais plus étudier la medicine	2	INV
12.	J’aimerais devenir médecin, même si cela impliquait de privilégier mon travail à ma famille	1	
13.	J’arrêterais d’étudier s’il devenait évident qu’il n’y avait pas de travail ou de place d’interne après l’obtention du diploma	3	INV
14.	Je n’aurais pas choisi la médecine si cela impliquait de cumuler des dettes financières substantielles	3	INV
15.	Je serais prêt à repasser mon bac afin d’obtenir une meilleure note si cela était nécessaire pour étudier la medicine	2	

Sous-échelles: 1=Acceptation de faire des sacrifices, 2=Volonté d’étudier la médecine. 3=Persistance
